# Does perceived organization support moderates the relationships between work frustration and burnout among intensive care unit nurses? A cross-sectional survey

**DOI:** 10.1186/s12912-023-01180-5

**Published:** 2023-01-23

**Authors:** Ren Yanbei, Ma Dongdong, Liu Yun, Wu Ning, Qin Fengping

**Affiliations:** 1 Department of Cardiology, Qilu Hospital of Shandong University, 107 Wenhuaxi Road, Jinan, 250012 China; 2 Department of Nephrology, Qilu Hospital of Shandong University, 107 Wenhuaxi Road, Jinan, 250012 China

**Keywords:** Burnout, Work frustration, Perceived organizational support, ICU nurses, Moderating

## Abstract

**Background:**

Intensive care unit (ICU) nurses are at high risk of burnout and warranting attention. Existing literature found that work frustration was related to burnout, whilst perceived organization support influenced the association of work frustration with burnout. The purpose of this study was to investigate the relationship of work frustration and burnout among ICU nurses, and to examine the moderating effect of perceived organization support in their relationship.

**Methods:**

The cross-sectional study was conducted with a convenience sample of 479 ICU nurses from several 3 tertiary hospitals during December 2021 to May 2022. The Maslach Burnout Inventory-Human services survey (MBI-HSS), National Aeronautics and Space Administration Task Load Index (NASA-TLX) and perceived organization support Scale (POSS) were used to collect data. The PROCESS macro was performed to test the moderation effect of perceived organization support.

**Results:**

The total score of burnouts was (55.79 ± 17.20), the total score of work frustration was (7.44 ± 1.86). Burnout was positively correlated with work frustration (*r* = 0.301, *P* < 0.001) and negatively correlated with perceived organizational support (*r* = -0.430, *P* < 0.001). The moderation model analysis showed that perceived organizational support could moderate the relationship between work frustration and burnout (β = -0.111, ΔR2 = 0.011, *P* = 0.007).

**Conclusions:**

The findings highlight the moderating role of perceived organizational support in the relationship between work frustration and burnout. Hence, interventions to reduce burnout among ICU nurses should consider targeting organizational support and work frustration.

**Supplementary Information:**

The online version contains supplementary material available at 10.1186/s12912-023-01180-5.

## Introduction

Burnout is generally described as a long-term response to unmanageable work stress and a syndrome of cynicism and professional ineffectiveness, characterized by high sense of emotional exhaustion and depersonalization and low sense of personal accomplishment [1]. Although nurses in different units generally reported various levels of burnout, nurses working in intensive care unit (ICU) always experienced it more remarkably. ICU nurses were often reported to be at high risk of burnout due to high-stress work environment, including heavy workload, insufficient nursing in workplace, disproportionate care, facing the continuous suffering of patients, and observing end-of-life and death [2, 3]. Empirical Studies have reported that the overall prevalence of burnout risk among ICU nurses was as high as 68% [4, 5]. Meanwhile, a systematic review also reported that the prevalence rate was highest among ICU nurses [6]. Moreover, long-term and serious burnout in nurses had been reported to be associated with job performance, physical and mental health and well-being, eventually contributing to decreased quality of care and increased turnover rate and exacerbating the shortage of nursing staff [7–9].

Previous studies have revealed several demographic and work-related variables linked to burnout among ICU nurses, such as age, gender, marital status, income satisfaction, work experience, professional title, and night shift [3–5]. For instance, older nurses were shown to experience less burnout [3], and male nurses reported a higher prevalence of burnout than did female nurses [3, 4]. ICU nurses that work in night shifts have reported higher levels of burnout [5]. However, there are not always consistent results on the associations of demographic and work-related variables with burnout among nurses [10]. For example, Bruyneel and colleagues found that older nurses were shown to experience more emotional exhaustion [4]. Given the high prevalence and negative effects of burnout among ICU nurses, in order to properly address the problem of burnout among ICU nurses, it is a crucial first step to fully understand the related factors of burnout among ICU nurses so that effective interventions can be developed to improve the motivations of nurses for working and the quality of health care.

The job demands–resources (JD-R) model proposes two relatively independent processes that job demands and resources may evoke health impairment and employee motivation, respectively [11]. This model suggests that a high level of job demands may result in employee experiencing physical and mental workloads and burnout, while job resources initiate a motivational process leading to positive organizational outcomes, including enhanced performance and work engagement [12]. Nursing is a stressful and challenging occupation [13]. Challenges encountered by nurses working in ICU were not only related to high prevalence of burnout risk, but also identified as the leading causes of work frustration for nurses [14–16]. Work frustration refers to a negative work affect generated by exhausted motivation and unsatisfied needs resulting from the obstacles and interference encountered by individuals in the workplace, which had been conceptualized as one of job demands [17, 18] and considered as the precursor to burnout [19, 20]. Previous studies have reported that work frustration was commonly in nurses due to a sense of being disrespected, long work hours, effort–reward imbalance, and issues in team cooperation, which were identified as the mainly factors causing work frustration for nurses [14, 21, 22]. Meanwhile, such negative emotion reaction has been reported to be positive correlation with nursing personnel’s emotional depletion, turnover intention, and professional commitment [19, 21, 22]. However, the association of work frustration with burnout has seldom been investigated among ICU nurse. Given the high risk and adverse effect of work frustration, the association between work frustration and burnout among ICU nurses requires thorough analysis.

Perceived organizational support, as a valued job resource, refers to employees’ evaluation of the extent to which the organization help, affirmation and concern about their presence in the organization. According to theoretical and empirical evidence, perceived organizational support can produce a sense of responsibility and obligation to help the organization achieve goals, foster employee’s enthusiastic and positive work attitude [23, 24]. Moreover, perceived organizational support acts as an effectively contextual resource had been confirmed that could influence the effects of emotional labor, work strain and workplace ostracism on job-related outcomes [25, 26]. Previous studies had demonstrated that the moderating impact of perceived organizational support on the relationship between job stress and job-related outcomes based on surveys of non-nurses [27, 28]. Subsequent researches based on nurses have also found that perceived organizational support could moderate the relationships between emotional labor and work attitudes [29] and the association of resilience with fatigue [30]. Accordingly, perceived organization support could act as a moderator on the association of work frustration with burnout among ICU nurses, which has not been reported and needs further verification.

In light of the conceptual frameworks and practical concerns, the present study aimed to examine two hypotheses in Chinese nurses: (1) work frustration could be positively associated with burnout, and (2) perceived organization support could moderate the direct association between work frustration and burnout.

## Method

### Participants

This study was a cross-sectional design and adhered to the STROBE statement. A convenience sample of ICU nurses was recruited from several tertiary hospitals in urban areas of Jinan, China between December 2021 and May 2022. The sample size was calculated as 462 based on the formula: N = (Z_α/2_)^2^P(1-P)/δ^2^ and a twenty percent attrition rate [31]. The assumptions were that α = 0.05, Z_α/2_ = 1.96, and δ = 0.05, whereas P was set as 0.5 due to large differences in the prevalence of burnout in nurses reported in previous studies and availability of maximum sample size. All ICU nurses who had obtained professional certificates and were independently responsible for clinical work. The exclusion criteria were as follows: (1) ICU nurses in departments with fewer than eight beds, (2) nurses who had worked less than one year, (3) nurses who were on vacation or going on leave to study, and (4) nurses who worked in both the ICU and the wards at the same time. ICU that had less than eight critical care beds typically do not care for ventilated patients for more than 24 h. Besides, according to guidelines for intensive care unit (ICU) construction and management in China 2006 edition [32], the recommended number of ICU beds for a tertiary hospital is no less than 8. Hence, ICU nurses in departments with fewer than eight beds were excluded. Ethical approval was obtained from the Research Ethics Committee of Qilu Hospital of Shandong University (KYLL-202107–031). Written informed consent was obtained from all participants. The procedures were conducted per the ethical standards of the 1964 Declaration of Helsinki. A total of 490 ICU nurses who met the inclusion criteria and were invited to participate in this study. After eliminating incomplete questionnaires, 479 ICU nurses remained for analysis. A comparison of the 11 excluded nurses with the 479 included participants found no significant differences in the socio-demographic variables.

## Measures

A self-administered, structured questionnaire including instruments for assessing socio-demographic variables, work frustration, perceived organization support and burnout was used to collect data.

### Demography

The socio-demographic questionnaire was designed by the authors and included participant’s age, gender, marital status, educational, income satisfaction, work experience, professional title, and night shift.

### Work frustration

One item selected from the National Aeronautics and Space Administration Task Load Index (NASA-TLX) was used to assess nurses’ perception of work frustration. The NASA-TLX is primarily a measure of how an individual experiences the situational demands of work [33, 34]. It consists of 6 items that evaluates six dimensions regarding different aspects of workload, including mental demands, physical demands, temporal demands, performance, effort and frustration. Scores for each item ranged from 0 (low) to 10 (high), and with higher scores indicating more workload. The validity and reliability of NASA-TLX have been confirmed in previous studies [35, 36]. In this study, only one item was adopted for the survey and results analysis. The translated item as follows: “How insecure, discouraged, irritated, stressed and annoyed versus secure, gratified, content, relaxed and complacent did you feel during your work?”. Responses are rated from 0 (low) to 10 (high), and with higher scores indicating more work frustration.

### Perceived organization support

The 8-item Chinese vision of the Survey of perceived organization support [37, 38] was used to assess nurses’ perception that the organization valued their contribution and cared about their well-being. Respondents indicated the extent of their agreement with each item on a 7-point Likert-type scale (1 = strongly agree, 7 = strongly disagree), and with higher scores indicating high perception of organizational support. The validity and reliability have been confirmed among Chinese occupational groups in previous studies [39, 40]. In this study, the Cronbach’s alpha for this scale was 0.887.

### Burnout

The Chinese version of Maslach Burnout Inventory-Human services survey (MBI-HSS) was used to measure nurse’s burnout [41, 42]. The MBI-HSS consists of 22 items that evaluates the three components of the burnout syndrome: emotional exhaustion (9 items), depersonalization (5 items), and personal accomplishment (8 items). The respondents were asked to indicate their frequency of experience on a 7-point Likert scale (0 = feeling has never been experienced, 6 = feeling is experienced daily). The items score of personal accomplishment have been reverse coded so that higher scores represent diminished personal accomplishment. The higher total scores of the three subscales means high level of burnout [43, 44], and the distribution data in each subscale were also provided. The validity and reliability have been confirmed among nurses in Chinese [45, 46]. In this study, the Cronbach’s alpha for of the total and its three sub-dimensions were 0.878, 0.883, 0.801 and 0.862, respectively. Permission to use the MBI-HSS which is copyrighted was obtained from Mind Garden.

### Data analysis

Data analysis was conducted by SPSS version 26.0 (IBM Corp., 2019). Mean, standard deviations or frequency, percentages were used to describe the characteristics of participants. Independent t test and analysis of variance analysis (ANOVA) were used to examine the differences in burnout and work frustration between sample characteristics. Pearson’s correlations were used to examine the associations among burnout, work frustration and perceived organizational support.

The moderation model was conducted using the SPSS PROCESS V3.5 macro developed by Hayes. Model 1 was used to examine the moderation role of organizational support on the effect of frustration on burnout [47]. The simple slope test by both pick-a-point method and the Johnson-Neyman method using the PROCESS macro were performed to test the significance of the moderation effect [48]. The covariates that were significantly associated with burnout in the univariate analyses had been adjusted for moderation model analyses. To avoid multi-collinearity effects, burnout, frustration and organizational support were standardized. *P* values reported were two tailed, and P value less than 0.05 was considered significant.

## Results

### Sociodemographic characteristics and Univariate analyses

The demographic characteristics of ICU nurses were presented in Table 1. The mean age of ICU nurses was (29.67 ± 4.76) years, and the mean working experience were (7.01 ± 5.56) years. The scores of work frustration was (7.44 ± 1.86), and the total scores of burnout was (55.79 ± 17.20), including emotional exhaustion score was (28.94 ± 10.32), depersonalization score was (10.15 ± 6.65) and diminished personal accomplishment score was (16.70 ± 8.58). There were significant differences in work frustration between income satisfaction groups. In addition, there were significant differences in burnout between groups in term of age, marital status, income satisfaction, working experiences and professional title.

**Table1:** Sociodemographic characteristics and Univariate analyses (*n* = 479)

Variable	n (%)	work frustration (M ± SD)	*t/ F (P)*	job burnout (M ± SD)	*t/ F (P)*
Age	29.67 ± 4.76		*F* = 1.026		*F* = 1.934
≤ 25 ^a^	80(16.7)	7.68 ± 1.90	(0.381)	58.51 ± 16.48	(0.123)
26–30 ^b^	226(47.2)	7.45 ± 1.84		56.34 ± 17.44	a > d
31–35 ^c^	134(28.0)	7.25 ± 1.90		54.60 ± 16.32	
> 35 ^d^	39(8.1)	7.62 ± 1.80		51.10 ± 19.41	
Gender			*t* = 0.358		*t* = 0.705
male	97(20.3)	7.51 ± 1.94	(0.721)	56.49 ± 18.05	(0.651)
female	382(79.7)	7.43 ± 1.84		55.61 ± 16.99	
Marital status			*t* = 0.165		*t* = 2.185
married	344(71.8)	7.45 ± 1.85	(0.869)	54.72 ± 17.10	(0.029)
single or others	135(28.2)	7.42 ± 1.91		58.52 ± 17.21	
Education			*t* = 1.446		*t* = 0.931
College and below	115(24.0)	7.23 ± 1.80	(0.149)	54.49 ± 16.53	(0.352)
Undergraduate and above	364(76.0)	7.51 ± 1.88		56.20 ± 17.40	
Income satisfaction			*F* = 11.006		*F* = 20.794
Satisfaction ^a^	73(15.2)	7.05 ± 1.91 ^a^	(< 0.001)	47.32 ± 16.76	(< 0.001)
General ^b^	257(53.7)	7.22 ± 1.78 ^b^	a/b < c	54.60 ± 17.41	a < b < c
Dissatisfaction ^c^	149(31.1)	8.02 ± 1.87 ^c^		62.00 ± 14.75	
Work Experience(years)	7.01 ± 5.56		*F* = 1.388		*F* = 1.813
≤ 2 ^a^	82(17.1)	7.57 ± 1.91	(0.246)	59.37 ± 16.21	(0.144)
2–5 ^b^	143(29.9)	7.43 ± 1.83		56.08 ± 17.22	a > d/c
5–10 ^c^	186(38.8)	7.27 ± 1.85		54.85 ± 16.81	
> 10 ^d^	68(14.2)	7.78 ± 1.88		53.44 ± 18.96	
Professional title			*t* = 0.438		*t* = 2.441
Nurse practitioner	389(81.2)	7.43 ± 1.83	(0.661)	56.71 ± 16.90^b^	(0.015)
Nurse-in-charge	83(18.8)	7.52 ± 1.99		51.82 ± 17.99 ^c^	
Night shift			*t* = 0.034		*t* = 1.369
yes	449(93.7)	7.45 ± 1.87	(0.973)	56.07 ± 17.06	(0.172)
no	30(6.3)	7.43 ± 1.83		51.53 ± 18.89	

*M* mean, *SD* standard deviation, *t* independent t-test, *F* analysis of variance

Further analysis of differences in three subscales of burnout between sociodemographic characteristics showed in Supplementary Table S1. The results revealed that there were significant differences in depersonalization between groups in term of age, marital status, income satisfaction, working experiences and professional title. In addition, there were also significant differences in emotional exhaustion and diminished personal accomplishment between income satisfaction groups.

### Correlational analyses

Mean and standard deviations for the variables and correlations between the variables were shown in Table 2. Burnout was positively associated with work frustration among (*r* = 0.301, *P* < 0.001) and negatively associated with perceived organizational support (*r* = -0.430, *P* < 0.001). Besides, work frustration was negatively associated with perceived organizational support (*r* = -0.281, *P* < 0.001).

**Table 2:** Inter-correlations of main study variables (*n* = 479)

Variable	Rate range	M ± SD	1	1.1	1.2	1.3	2
1. Burnout	9–97	55.79 ± 17.20	1				
1.1 emotional exhaustion	6–53	28.94 ± 10.32	0.767^***^	1			
1.2 depersonalization	0–30	10.15 ± 6.65	0.794^***^	0.591^***^	1		
1.3 diminished personal accomplishment	0–40	16.70 ± 8.58	0.466^***^	0.223^***^	0.206^***^	1	
2. Work frustration	3–10	7.44 ± 1.86	0.301^***^	0.406^***^	0.224^***^	0.158^**^	1
3. POS	8–56	37.04 ± 7.88	-0.430^***^	-0.413^***^	-0.254^***^	-0.170^***^	-0.281^***^

*M* mean, *SD* standard deviation, *POS* Perceived organizational support

^*^ *P* < 0.05

^**^ *P* < 0.01

^***^ *P* < 0.001

### Moderation analyses

**Fig. 1:**
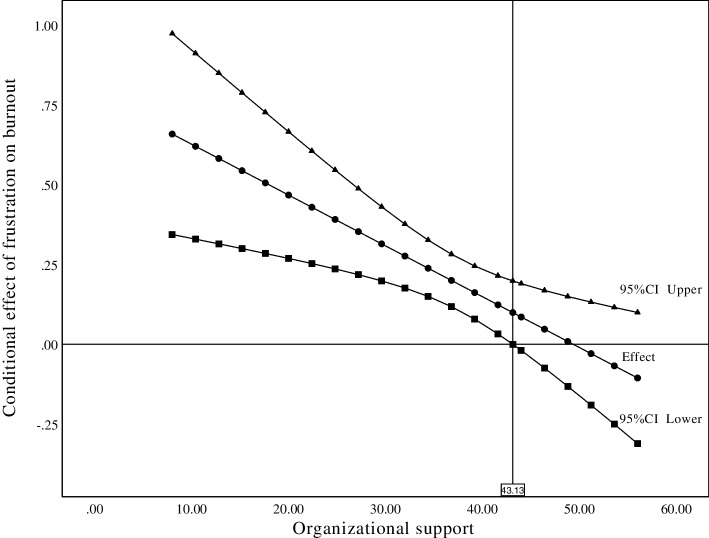
The conditional effect of work frustration on burnout at the values of perceived organizational support

The moderation analysis established whether perceived organizational support moderated the relationship between work frustration and burnout. The results presented in Table 3 and indicated that the interaction term of work frustration with perceived organizational support (*β* = -0.152, *P* = 0.007) significantly accounted for 1.1% variance of burnout with a small effect size (*f*^2^ = 0.015). The pick-a-point method (Table 4) indicated that the negative effects of work frustration on burnout decreased as perceived organizational support increased under three different levels. The results of the Johnson-Neyman method (Fig. 1) demonstrated that significant conditional effect of work frustration on burnout was found when perceived organizational support was lower than 43.10.

**Table 3:** Moderation model (*n* = 479)

Variable	β	SE	t	P	95%CI
Age	0.018	0.023	0.782	0.435	-0.027 to 0.062
Marital status	-0.155	0.104	-1.492	0.136	-0.359 to 0.049
Satisfaction of income	0.172	0.064	2.660	0.008	0.045 to 0.298
Years of working	-0.043	0.021	-2.075	0.039	-0.083 to -0.002
Professional title	0.109	0.087	1.257	0.210	-0.062 to 0.281
Work frustration	0.194	0.042	4.667	< 0.001	0.113 to 0.276
POS	-0.305	0.046	-6.697	< 0.001	-0.394 to -0.215
Interaction term	-0.111	0.041	-2.695	0.007	-0.193 to -0.030

Interaction term means the interaction of work frustration and perceived organizational support

*SE* standard error, *CI* confidence interval, *POS* Perceived organizational support

**Table 4:** Conditional effects of work frustration on job burnout at different levels of organizational support (*n* = 479)

Conditional Of POS	Effect	SE	t	p	95%CI
M-1SD	0.306	0.061	5.041	< 0.001	0.187 to 0.425
M	0.194	0.042	4.667	< 0.001	0.113 to 0.276
M + 1SD	0.083	0.057	1.466	0.144	-0.028 to 0.194

*POS* Perceived organizational support, *SE* standard error, *CI* confidence interval, *M* mean, *SD* standard deviation

Similarly, moderation analysis examined whether perceived organizational support moderated the relationships between work frustration and three subscales of burnout. The results showed that moderating effect of perceived organizational support was only found in relationship between work frustration and emotional exhaustion. The results of moderation model were also presented in Supplementary Table S2. The interaction term of work frustration with perceived organizational support (β =  − 0.170, *P* = 0.002) significantly accounted for 1.8% variance of emotional exhaustion with a small effect size (*f*^2^ = 0.03). The pick-a-point method (Supplementary Table S3) indicated that the positive effects of work frustration on emotional exhaustion decreased as perceived organizational support increased under three different levels. The results of the Johnson-Neyman method (Supplementary Fig. S1) demonstrated that significant conditional effect of work frustration on emotional exhaustion was found when perceived organizational support was lower than 47.66.

## Discussion

This is the first study to explore the associations between work frustration and burnout, and examine the moderating role of perceived organization support between them among ICU nurses. The results indicated that ICU nurses with high work frustration and low perceived organization support had more burnout. Moreover, the results from the moderation analysis showed that perceived organization support moderated the associations between work frustration and burnout as well as emotional exhaustion among ICU nurses.

In the present study, 46.2% of ICU nurses with work frustration scores greater than 7 can be considered to have high level of work frustration, which was higher than Wang et al. survey of senior nurses in Taiwan [22]. The mean score of burnout was similar to previous studies conducted in ICU nurses [49, 50]. The results might indicate that the high level of work frustration could be significant factor of higher risk of burnout among ICU nurses [6]. In addition, ICU nurses who had lower income satisfaction were more likely to report higher level of work frustration and burnout, which were in line with previous findings [51, 52]. The lower income satisfaction might reflect work with imbalanced extrinsic effort and reward, which signifies a failed social reciprocity that elicits work stress [30, 53]. Furthermore, ICU nurses who had shorter work experience were more likely to report high level of burnout, as well as higher depersonalization. In accordance with prior studies, increasing work experience gradually increases more professional maturity, which in turn may keep control during times of stress [54, 55].

As hypothesized, ICU nurses who had perceived higher level of work frustration and lower level of organizational support experienced higher level of burnout, which were similar to previous studies [20, 56]. As one of job demands, work frustration refers to the situational feeling of disappointment and dissatisfaction toward work. For ICU nurses, work frustration may derive from various challenges, including heavy workload, insufficient nursing in workplace, disproportionate care, facing the continuous suffering of patients, and observing end-of-life and death [2, 3]. It was plausible that ICU nurses who had perceived higher level of work frustration could feel blockage the opportunity of achieving valued goals and needs in job, which could lead to emotional drain and burnout [57]. There were many reports on relationships between perceived organization support and burnout. A prior systematic review by Almudena et al. concluded that perceived organization support, as an external source of work, could help reduce job stress and burnout among nurses [58]. ICU nurses who perceived low perceived organization support might often receive insufficient material and emotional support from the organization and then lead to unmet socioemotional needs and stressful work environment [59], which might increase nurses’ burnout.

This study confirmed the moderating role of perceived organization support in the relationships between work frustration and burnout as well as emotional exhaustion, that is, organizational support could buffer the impact of work frustration on burnout. Specifically, the effect of work frustration on burnout as well as emotional exhaustion were increased among ICU nurses with lower perceived organization support and attenuated in those with higher perceived organization support. The possible explanation for this association might be that perceived organization support could influence individuals’ stress appraisal and their perception of available stress-coping resources by its four typical functions [60], namely maintaining and promoting self-esteem, providing information, providing social companionship and providing material resources, and ultimately contributed to lower burnout among ICU nurses. Besides, according to the results of Johnson-Neyman test, the associations of work frustration and burnout as well as emotional exhaustion was not significant when perceived organization support increased to some extent, which might implicate the potential value of improving organizational support in reducing burnout among ICU nurses.

This study advances the current state of knowledge by examining the relationships between work frustration and burnout among ICU nurses. Most importantly, this study contributes to the evidence by testing the moderating effect of perceived organization support on the relationship between work frustration and burnout among ICU nurses. This study has important practical implications for reducing and prevent ICU nurses’ burnout. Based on the findings of the present study, lower income satisfaction was most consistent factor related to high level of work frustration and burnout. Hence, it is recommended for nursing administrators that salary system reform could be made to improve income and benefits among ICU nurses. Besides, nursing administrators should consider providing effective and targeted strategies (e.g. ongoing training and psychological interventions) to improve the conditions of their working environments and decrease their work frustration among ICU nurses. Perceived organization support was not only related to low level of burnout, but could also buffer the negative effect of work frustration on burnout. Thus, it could reduce ICU nurses’ burnout by providing nurses with organizational supports through demonstrating publicly to ICU nurse that the organization cares about their welfare, values their opinions, and is proud of their achievements [26].

Despite these Strengths, several limitations should be mentioned. First, due to the cross-sectional study design and small sample size, the causal inference among study variables and the generalizability of the results are limited. Hence, future research can be improved by longitudinal studies with larger multicenter sample size. Second, a single-item measurement was adopted to capture work frustration, which may limit the validity of the study findings. Future studies that employ a more comprehensive measurement of work frustration would further facilitate the understanding of the relationships addressed in this study. Third, the size of interaction effect detected is small (*f*^2^ = 0.015) in this study. Further validation by large sample size is warranted. In addition, self-reported results might be subject to information bias.

## Conclusion

The high level of work frustration and burnout were experienced among ICU nurses, especially who had lower income satisfaction and shorter work experience. Work frustration was found to be associated with increased burnout among ICU nurses. Moreover, perceived organization support played a mild moderating role in associations of work frustration with burnout in this group. Thus, future interventions seeking to reduce burnout in this group should be considered to decrease work frustration and tailored to ICU nurses with varying perceived organization support.

## Supplementary Information

**Additional file 1: Table S1. **Differences in emotional exhaustion, depersonalization,and diminished personal accomplishment among ICU nurses (*n*=479). **Table S2.** Moderation model (*n*=479). **Table S3.** Conditional effects of work frustration on emotional exhaustionat different levels of organizational support (*n*=479). **Fig.S1.** The conditional effect of work frustration on emotional exhaustion at thevalues of perceived organizational support.  

## Data Availability

The datasets analyzed during the current study are not publicly available due to them containing information that could compromise research participant consent but are available from the corresponding author on reasonable request.

## Abbreviations

ICU: Intensive care unit
MBI: Maslach burnout inventory
NASA-TLX: National aeronautics and space administration task load index
POSS: Perceived organization support Scale

## References

[1] Maslach C, Schaufeli WB, Leiter MP (2001). Job burnout. Annu Rev Psychol.

[2] Chuang CH, Tseng PC, Lin CY, Lin KH, Chen YY (2016). Burnout in the intensive care unit professionals: a systematic review. Medicine (Baltimore).

[3] Ramírez-Elvira  S, Romero-Béjar  JL,  Suleiman-Martos  N, Gómez-Urquiza  JL , Monsalve-Reyes  C , Cañadas-De la Fuente  GA, Albendín-García  L (2021). Prevalence, risk factors and burnout levels in intensive care unit nurses: a systematic review and meta-analysis. Int J Environ Res Public Health.

[4] Bruyneel A, Smith P, Tack J, Pirson M (2021). Prevalence of burnout risk and factors associated with burnout risk among ICU nurses during the COVID-19 outbreak in French speaking Belgium. Intensive Crit Care Nurs.

[5] Hu Z, Wang H, Xie J, Zhang J, Li H, Liu S, Li Q, Yang Y, Huang Y (2021). Burnout in ICU doctors and nurses in mainland China-a national cross-sectional study. J Crit Care.

[6] Woo T, Ho R, Tang A, Tam W (2020). Global prevalence of burnout symptoms among nurses: a systematic review and meta-analysis. J Psychiatr Res.

[7] Salyers MP, Fukui S, Rollins AL, Firmin R, Gearhart T, Noll JP, Williams S, Davis CJ (2015). Burnout and self-reported quality of care in community mental health. Adm Policy Ment Health.

[8] Alenezi A, McAndrew S, Fallon P (2019). Burning out physical and emotional fatigue: evaluating the effects of a programme aimed at reducing burnout among mental health nurses. Int J Ment Health Nurs.

[9] Maslach C, Leiter MP (2016). Understanding the burnout experience: recent research and its implications for psychiatry. World Psychiatry.

[10] Cañadas-De la Fuente  GA, Ortega  E, Ramirez-Baena  L,  De la Fuente-Solana EI, Vargas C, Gómez-Urquiza  JL (2018). Gender, marital status, and children as risk factors for burnout in nurses: a meta-analytic study. Int J Environ Res Public Health.

[11] Demerouti E, Bakker AB, Nachreiner F, Schaufeli WB (2001). The job demands-resources model of burnout. J Appl Psychol.

[12] Bakker AB, Demerouti E, Euwema MC (2005). Job resources buffer the impact of job demands on burnout. J Occup Health Psychol.

[13] Alfuqaha O, Alsharah H (2018). Burnout among nurses and teachers in Jordan: a comparative study. Arch Psychiatry Psychother.

[14] Chang YP, Lee DC, Wang HH (2018). Violence-prevention climate in the turnover intention of nurses experiencing workplace violence and work frustration. J Nurs Manag.

[15] Moradi Y, Baghaei R, Hosseingholipour K, Mollazadeh F (2021). Challenges experienced by ICU nurses throughout the provision of care for COVID-19 patients: a qualitative study. J Nurs Manag.

[16] Jarden RJ, Sandham M, Siegert RJ, Koziol-McLain J (2019). Strengthening workplace well-being: perceptions of intensive care nurses. Nurs Crit Care.

[17] Bakker AB, Demerouti E, Verbeke W (2004). Using the job demands-resources model to predict burnout and performance. Hum Resour Manage.

[18] Ntsiful A, Ahiakpor L, Damoah J, Wee GSM (2018). Frustration at work, developmental experience, perceived team support and employee performance: evidence from emerging economies. Pan-Afr J Bus Manag.

[19] Chang YP, Wang HH, Huang S, Wang HI (2014). Interaction effect of work excitement and work frustration on the professional commitment of nurses in Taiwan. J Nurs Res.

[20] Lewandowski CA (2003). Organizational factors contributing to worker frustration: the precursor to burnout. J Soc Soc Welf.

[21] Lin Y-Y, Lee Y-H, Chang S-C, Lee D-C, Lu K-Y, Hung Y-M, Chang Y-P (2019). Individual resilience, intention to stay, and work frustration among postgraduate two-year programme nurses. Collegian.

[22] Wang PH, Ku YC, Chen CC, Jeang SR, Chou FH (2016). Work-related frustration among senior nurses at a medical centre. J Clin Nurs.

[23] Byrne ZS, Hochwarter WA (2008). Perceived organizational support and performance. J Manag Psychol.

[24] Kurtessis JN, Eisenberger R, Ford MT, Buffardi LC, Stewart KA, Adis CS (2015). Perceived organizational support: a meta-analytic evaluation of organizational support theory. J Manag.

[25] Sarfraz M, Qun W, Sarwar A, Abdullah MI, Imran MK, Shafique I (2019). Mitigating effect of perceived organizational support on stress in the presence of workplace ostracism in the Pakistani nursing sector. Psychol Res Behav Manag.

[26] Xu Z, Yang F (2021). The impact of perceived organizational support on the relationship between job stress and burnout: a mediating or moderating role?. Curr Psychol.

[27] Lai H, Hossin MA, Li J, Wang R, Hosain MS (2022). Examining the relationship between covid-19 related job stress and employees' turnover intention with the moderating role of perceived organizational support: evidence from SMEs in China. Int J Environ Res Public Health.

[28] Bao Y, Zhong W (2019). How stress hinders health among chinese public sector employees: the mediating role of emotional exhaustion and the moderating role of perceived organizational support. Int J Environ Res Public Health.

[29] Lartey JKS, Amponsah-Tawiah K, Osafo J (2019). The moderating effect of perceived organizational support in the relationship between emotional labour and job attitudes: a study among health professionals. Nurs Open.

[30] Liu L, Wu D, Wang L, Qu Y, Wu H (2020). Effort-reward imbalance, resilience and perceived organizational support: a moderated mediation model of fatigue in chinese nurses. Risk Manag Healthc Policy.

[31] Serdar CC, Cihan M, Yücel D, Serdar MA (2021). Sample size, power and effect size revisited: simplified and practical approaches in pre-clinical, clinical and laboratory studies. Biochem Med (Zagreb).

[32] Yu K, Ma X, Fang Q, Liu D, An Y, Qiu H, Yan J, Qin T, Guan X, Kang Y (2006). Guidelines for intensive care unit (ICU) construction and management in China (2006). Chin J Surg.

[33] Hart SG, Staveland LE. Development of NASA-TLX (Task Load Index): Results of empirical and theoretical research. In: Human mental workload. edn. Oxford, England: North-Holland; 1988: 139–183.

[34] Xiao Y, Wang Z, Wang M, Lan YJ (2005). The appraisal of reliability and validity of subjective workload assessment technique and NASA-task load index. Chin J Ind Hyg Occup Dis.

[35] Hoonakker P, Carayon P, Gurses A, Brown R, McGuire K, Khunlertkit A, Walker JM (2011). Measuring workload of ICU nurses with a questionnaire survey: the NASA Task Load Index (TLX). IIE Trans Healthc Syst Eng.

[36] Tubbs-Cooley HL, Mara CA, Carle AC, Gurses AP (2018). The NASA Task Load Index as a measure of overall workload among neonatal, paediatric and adult intensive care nurses. Intensive Crit Care Nurs.

[37] Eisenberger R, Huntington R, Hutchison S, Sowa D (1986). Perceived organizational support. J Appl Psychol.

[38] Eisenberger R, Cummings J, Armeli S, Lynch P (1997). Perceived organizational support, discretionary treatment, and job satisfaction. J Appl Psychol.

[39] Tao Y, Ma Z, Hou W, Zhu Y, Zhang L, Li C, Shi C (2022). Neuroticism trait and mental health among chinese firefighters: the moderating role of perceived organizational support and the mediating role of burnout-a path analysis. Front Public Health.

[40] Shen J, Benson J (2014). When CSR is a social norm: how socially responsible human resource management affects employee work behavior. J Manag.

[41] Maslach C, Jackson SE, Leiter MP. Maslach Burnout Inventory: Third edition. In: Evaluating stress: A book of resources. edn. Lanham, MD, US: Scarecrow Education; 1997: 191–218.

[42] Yuen M, Lau PSY, Shek DTL, Lam M-P (2002). Confirmatory factor analysis and reliability of the Chinese version of the Maslach burnout inventory among guidance teachers in Hong Kong. Psychol Rep.

[43] Anwar MM, Elareed HR (2017). Burnout among Egyptian Nurses. J Public Health.

[44] Zakeri MA, Rahiminezhad E, Salehi F, Ganjeh H, Dehghan M (2021). Burnout, anxiety, stress, and depression among iranian nurses: before and during the first wave of the COVID-19 Pandemic. Front Psychol.

[45] Lee HF, Chien TW, Yen M (2013). Examining factor structure of Maslach Burnout Inventory among nurses in Taiwan. J Nurs Manag.

[46] Lin F, St John W, McVeigh C (2009). Burnout among hospital nurses in China. J Nurs Manag.

[47] Hayes AF. Introduction to mediation, moderation, and conditional process analysis: A regression-based approach: Guilford publications; 2017.

[48] Hayes AF, Matthes J (2009). Computational procedures for probing interactions in OLS and logistic regression: SPSS and SAS implementations. Behav Res Methods.

[49] Shbeer A, Ageel M (2022). Assessment of occupational burnout among intensive care unit Staff in Jazan, Saudi Arabia, using the maslach burnout inventory. Crit Care Res Prac.

[50] Xie C, Li X, Zeng Y, Hu X (2021). Mindfulness, emotional intelligence and occupational burnout in intensive care nurses: a mediating effect model. J Nurs Manag.

[51] Xie W, Wang J, Zhang Y, Zuo M, Kang H, Tang P, Zeng L, Jin M, Ni W, Ma C (2021). The levels, prevalence and related factors of compassion fatigue among oncology nurses: a systematic review and meta-analysis. J Clin Nurs.

[52] See KC, Zhao MY, Nakataki E, Chittawatanarat K, Fang WF, Faruq MO, Wahjuprajitno B, Arabi YM, Wong WT, Divatia JV (2018). Professional burnout among physicians and nurses in Asian intensive care units: a multinational survey. Intensive Care Med.

[53] Padilla Fortunatti C, Palmeiro-Silva YK (2017). Effort-reward imbalance and burnout among ICU Nursing Staff: a cross-sectional study. Nurs Res.

[54] Demir A, Ulusoy M, Ulusoy MF (2003). Investigation of factors influencing burnout levels in the professional and private lives of nurses. Int J Nurs Stud.

[55] Nobre DFR, Rabiais ICM, Ribeiro P, Seabra PRC (2019). Burnout assessment in nurses from a general emergency service. Rev Bras Enferm.

[56] Potard C, Landais C (2021). Relationships between frustration intolerance beliefs, cognitive emotion regulation strategies and burnout among geriatric nurses and care assistants. Geriatr Nurs.

[57] Ogungbamila B, Olusa AO (2016). Job-related frustration, work-family interference, and occupational burnout: suppressive roles of perceived family supportiveness and emotional intelligence. Int J Educ Manag Stud.

[58] Velando‐Soriano  A, Ortega‐Campos  E,  Gómez‐Urquiza  JL , Ramírez‐Baena L, De La Fuente  EI, Cañadas‐De La Fuente  GA (2020). Impact of social support in preventing burnout syndrome in nurses: a systematic review. Jpn J Nurs Sci.

[59] Rhoades L, Eisenberger R (2002). Perceived organizational support: a review of the literature. J Appl Psychol.

[60] Cohen S, Wills TA (1985). Stress, social support, and the buffering hypothesis. Psychol Bull.

