# Supplementary material for: Does perceived organization support moderates the relationships between work frustration and burnout among intensive care unit nurses? A cross-sectional survey
Source: BMC Nurs. 2023 Jan 23;22:22. doi: 10.1186/s12912-023-01180-5 (PMC9872303; doi:10.1186/s12912-023-01180-5)
Supplement: Supplementary file 1 — Additional file 1: Table S1. Differences in emotional exhaustion, depersonalization,and diminished personal accomplishment among ICU nurses (n=479). Table S2. Moderation model (n=479). Table S3. Conditional effects of work frustration on emotional exhaustionat different levels of organizational support (n=479). Fig.S1. The conditional effect of work frustration on emotional exhaustion at thevalues of perceived organizational support. [file 12912_2023_1180_MOESM1_ESM.docx]

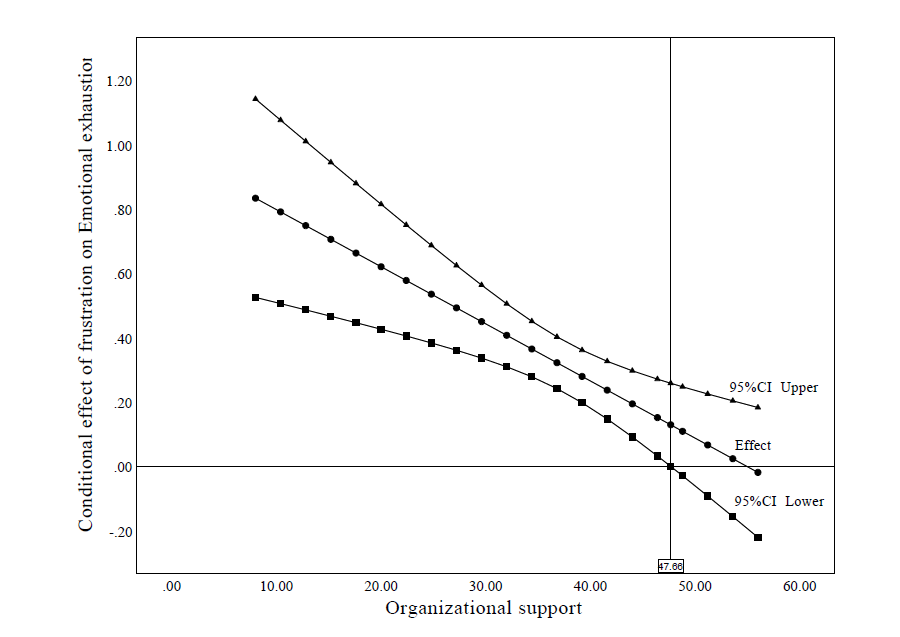


**Fig.S1**

**The conditional effect of work frustration on emotional exhaustion at the values of perceived organizational support.**

**Table S1. Differences in emotional exhaustion, depersonalization, and diminished personal accomplishment among ICU nurses (n=479)**

| Variable | n (%) | Emotional exhaustion (M±SD) | Depersonalization  (M±SD) | Diminished personal accomplishment (M±SD) |
| --- | --- | --- | --- | --- |
| Age | 29.67±4.76 |  | a>c/d, b>d |  |
| ≤25 ^a^ | 80(16.7) | 30.44±9.91 | 11.44±6.41 | 16.64±8.08 |
| 26-30 ^b^ | 226(47.2) | 29.21±10.50 | 10.53±6.73 | 16.59±8.76 |
| 31-35 ^c^ | 134(28.0) | 27.95±9.68 | 9.49±6.46 | 17.16±8.67 |
| ＞35 ^d^ | 39(8.1) | 27.69±12.00 | 7.62±6.58 | 15.79±8.51 |
| *F (P)* |  | 1.213(0.304) | 3.646(0.013) | 0.287(0.835) |
| Gender |  |  |  |  |
| male | 97(20.3) | 28.85±10.64 | 10.93±6.95 | 16.72±9.48 |
| female | 382(79.7) | 28.97±10.25 | 9.96±6.56 | 16.69±8.35 |
| *t (P)* |  | 0.103(0.918) | 1.288(0.198) | 0.034(0.973) |
| Marital status |  |  |  |  |
| married | 344(71.8) | 28.57±10.44 | 9.69±6.64 | 16.46±8.63 |
| single or others | 135(28.2) | 29.89±9.98 | 11.33±6.54 | 17.30±8.46 |
| *t (P)* |  | 1.260(0.208) | 2.434(0.015) | 0.972(0.332) |
| Education |  |  |  |  |
| College and below | 115(24.0) | 28.42±10.76 | 9.78±5.92 | 16.28±8.83 |
| Undergraduate and above | 364(76.0) | 29.10±10.18 | 10.27±6.86 | 16.83±8.51 |
| *t (P)* |  | 0.614(0.539) | 0.684(0.494) | 0.597(0.551) |
| Income satisfaction |  | a<b<c | a<b<c | a<b/c |
| Satisfaction ^a^ | 73(15.2) | 25.45±9.27 | 7.49±5.68 | 14.37±8.93 |
| General ^b^ | 257(53.7) | 28.10±10.23 | 9.80±6.54 | 16.70±8.74 |
| Dissatisfaction ^c^ | 149(31.1) | 32.10±10.18 | 12.07±6.76 | 17.83±7.94 |
| *F (P)* |  | 12.596(<0.001) | 13.019(<0.001) | 4.037(0.018) |
| Work Experience(years) | 7.01±5.56 |  | a>c/d, b>d |  |
| ≤2 ^a^ | 82(17.1) | 30.43±9.82 | 12.07±6.59 | 16.87±8.90 |
| 2-5 ^b^ | 143(29.9) | 28.92±10.60 | 10.62±6.56 | 16.54±8.53 |
| 5-10 ^c^ | 186(38.8) | 28.75±10.02 | 9.56±6.31 | 16.54±8.28 |
| ＞10 ^d^ | 68(14.2) | 27.72±11.09 | 8.49±7.27 | 17.24±9.26 |
| *F (P)* |  | 0.906(0.438) | 4.534(0.004) | 0.135(0.939) |
| - Professional title |  |  |  |  |
| Nurse practitioner | 389(81.2) | 29.16±10.35 | 10.59±6.56 | 16.96±8.56 |
| Nurse-in-charge | 83(18.8) | 28.01±10.18 | 8.28±6.74 | 15.53±8.63 |
| *t (P)* |  | 0.949(0.171) | 2.994(0.003) | 1.427(0.154) |
| Shift-work |  |  |  |  |
| yes | 449(93.7) | 29.11±10.24 | 10.29±6.62 | 16.67±8.54 |
| no | 30(6.3) | 26.43±11.31 | 8.17±6.88 | 17.03±9.33 |
| *t (P)* |  | 1.377(0.169) | 1.694(0.091) | 0.223(0.824) |

M mean, SD standard deviation, *t* independent t-test, *F* analysis of variance.

**Table S2 Moderation model (n=479)**

| Dependent  variable | Independent variable | *β* | *SE* | *t* | *P* | *95%CI* |
| --- | --- | --- | --- | --- | --- | --- |
| Emotional exhaustion | Satisfaction of income | 0.086 | 0.063 | 1.374 | 0.170 | -0.037 to 0.209 |
|  | Work frustration | 0.318 | 0.041 | 7.784 | <0.001 | 0.238 to 0.394 |
|  | POS | -0.259 | 0.045 | 5.806 | <0.001 | -0.346 to -0.171 |
|  | Interaction term | -0.140 | 0.040 | 3.457 | <0.001 | -0.219 to -0.061 |
| Depersonalization | Age | 0.075 | 0.093 | 0.806 | 0.421 | -0.108 to 0.257 |
|  | Marital status | -0.141 | 0.122 | 1.161 | 0.246 | -0.380 to 0.098 |
|  | Satisfaction of income | 0.124 | 0.073 | 1.690 | 0.092 | -0.020 to 0.267 |
|  | Years of working | 0.049 | 0.814 | 0.605 | 0.546 | -0.111 to 0.209 |
|  | Professional title | -0.233 | 0.144 | 1.619 | 0.106 | -0.513 to 0.050 |
|  | Work frustration | -0.116 | 0.047 | 2.463 | 0.014 | -0.209 to -0.024 |
|  | POS | -0.165 | 0.051 | 3.215 | 0.001 | -0.266 to -0.064 |
|  | Interaction term | -0.024 | 0.047 | 0.516 | 0.606 | -0.116 to 0.068 |
| Diminished personal accomplishment | Satisfaction of income | 0.136 | 0.072 | 1.891 | 0.059 | -0.005 to 0.278 |
|  | Work frustration | -0.124 | 0.047 | 2.642 | 0.009 | -0.216 to -0.031 |
|  | POS | -0.168 | 0.051 | 3.275 | 0.001 | -0.269 to -0.067 |
|  | Interaction term | -0.019 | 0.047 | 0.417 | 0.677 | -0.111 to 0.278 |

SE standard error.CI confidence interval, POS Perceived organizational support.

Interaction term means the interaction of work frustration and perceived organizational support.

**Table S3 Conditional effects of work frustration on emotional exhaustion at different levels of organizational support (n=479)**

| Conditional Of POS | Effect | SE | t | p | 95%CI |
| --- | --- | --- | --- | --- | --- |
| M-1SD | 0.458 | 0.059 | 7.712 | <0.001 | 0.341 to 0.575 |
| M | 0.318 | 0.041 | 7.784 | <0.001 | 0.238 to 0.398 |
| M+1SD | 0.178 | 0.055 | 3.214 | 0.001 | 0.069 to 0.287 |

POS Perceived organizational support, SE standard error.CI confidence interval, M mean, SD standard deviation.
